# Underlying Dimensions of Borderline Personality Disorder: A Systematic Review of Factor Analytic Studies

**DOI:** 10.1007/s11126-025-10141-x

**Published:** 2025-04-05

**Authors:** Alexandra Triantafyllou, George Konstantakopoulos, Pentagiotissa Stefanatou, Eleni Giannouli, Ioannis A. Malogiannis

**Affiliations:** 1 First Department of Psychiatry, Eginition Hospital, Personality Disorders Unit, School of Medicine, National and Kapodistrian University of Athens, 11528 Athens, Greece; 2 Research Department of Clinical, Education and Health Psychology, University College London, London, WC1E 7HB UK; 3 Department of Speech and Language Therapy, University of Peloponnese, Kalamata, Greece

**Keywords:** Borderline personality disorder, Systematic review, Dimensions, Factor analysis

## Abstract

Borderline personality disorder (BPD) is considered to be a heterogeneous diagnostic entity, with multiple facets of the disorder influencing its course. Disentangling the structure of BPD criteria is fundamental to better understanding the disorder and targeting problematic behaviors. To identify and critically appraise factor analytic studies examining the structure of BPD criteria. A systematic review was conducted according to PRISMA guidelines. Three computerized databases (Pubmed, Scopus,PsycNET) were searched, resulting in the inclusion of 27 relevant studies. The unified model of BPD has been confirmed by a number of studies, while among multidimensional models, a three-factor model has also gained considerable empirical support. Multidimensional models of BPD tend to cluster together “disordered self” symptoms (identity disturbance and emptiness), affective symptoms (affective instability and anger), and behavioral symptoms (impulsivity and self-harming behaviors). Unidimensional and multidimensional models of the BPD structure are not necessarily in competition. An understanding of BPD as a unified diagnosis composed of three underlying dimensions, could serve the purpose of recognizing and targeting different aspects of the disorder, while maintaining a robust, and clinically useful diagnosis.

**Supplementary Information:**

The online version contains supplementary material available at 10.1007/s11126-025-10141-x.

## Introduction

Borderline personality disorder is a complex psychiatric disorder mainly characterized by impulsivity, emotional instability, identity disturbance and unstable relationships [1]. Heterogeneity seems to be at the core of the BPD construct since the term was first popularized by A. Stern [2]. Although the systematic work of researchers [3, 4] has led to a well-defined psychiatric diagnosis, the structure of the BPD criteria remains controversial. An important source of heterogeneity seems to be the nature of the criteria themselves, since, as mentioned by Sanislow & MacGlashan [5] they consist of a mixture of personality traits, symptomatic behaviors and symptoms. Akiskal [6] mentioned that the prevalence of various affective symptoms in BPD has contributed to the “unwieldy heterogeneity” of the disorder, leading him to argue strongly in favor of the view of BPD as an affective disorder. Tyrer [7], noting that BPD more than any other personality disorder consists of symptoms rather than traits, claims that BPD should not even be considered as a psychiatric disorder, as its very existence is “a passport to heterogeneity”. Another source of heterogeneity within the criteria, is the fact that BPD appears to lie on the border between internalizing and externalizing disorders [8–12], showing associations with both the distress subfactor of the internalizing dimension and the externalizing dimension [9]. To add to the complexity, any two individuals diagnosed with BPD may share only one common criterion, whereas 251 different combinations of criteria can lead to a BPD diagnosis [13]. Testing this hypothesis, Johansen, Karterud, Pedersen et al. [14] found that in a sample of 252 people diagnosed with BPD, 136 different combinations of criteria were formed, with the highest number of people sharing the exact criteria being 6. It should be noted that the ICD-10 [15] seemed to recognize the heterogeneity of emotionally unstable personality disorder by distinguishing between an impulsive and a borderline type. On the other hand, although the ICD-11 [16] adopts a dimensional trait-based diagnosis on personality disorders, retains the “borderline pattern” as a specifier.

The two main perspectives that researchers have taken in exploring BPD heterogeneity are a person-centered approach, usually using cluster analytic methods in the search of BPD subtypes, and a variable-oriented approach, which aims to unravel the structure of BPD criteria through factor analysis [17, 18]. Empirical studies on BPD subtyping have moved beyond the narrow confines of the psychiatric manual and have focused on identifying subgroups of people with BPD based on their ability to control and regulate emotions [19–21], on differences in experiencing and expressing anger, aggression and antisocial tendencies [18, 22, 23], on interpersonal functioning [24–27], on comorbidity with other personality disorders [28, 29], and on the differentiation of subtypes based on dimensions of borderline personality disorder[30–33]. While both perspectives of understanding borderline heterogeneity are of equal value, the present review focuses on the analysis of studies using a variable-centered approach, linking their findings to proposed subtypes.

From a variable-centered perspective, notable theoretical and research contributions to the borderline dimensions are those of Hurt, Clarkin, Widiger et al. [34], Linehan [35], Lieb, Zanarini, Schmahl et al. [36] and Gunderson [37]. Hurt et al. [34] assess the associational structure of the BPD criteria by identifying pairs of criteria that were most closely associated. Through pair clustering, three subsets of criteria emerged: an identity cluster, an affective cluster and an impulsive cluster. The research team concludes that meeting three of the criteria, one from each cluster, would successfully identify the majority of BPD individuals.

M. Linehan [35] organizes BPD criteria into five categories: affective dysregulation, interpersonal dysregulation, behavioral dysregulation, cognitive dysregulation, and disordered self. A few years later, she co-authored the article by Lieb et al. [36] who divided BPD criteria into four domains (affective, cognitive, behavioral and interpersonal area). An almost identical understanding of the structure of BPD dimensions is formulated by Gunderson [37], who, taking into account the existing empirical evidence suggesting the existence of underlying BPD dimensions, formulates an alternative proposal for the diagnosis of BPD, known as Gunderson’s algorithm. The algorithm suggests that five criteria from at least three out of the four sectors must be present in order for a BPD diagnosis to be made.

Oldham [38], in proposing his BPD subtypes, refers to the most prototypical criteria of each subtype, implying underlying dimensions of the borderline construct. Oldham [38] groups together the criteria of affective instability and recurrent suicidal behavior as the most prominent criteria of the affective type, impulsivity and suicidal/self-injurious behavior are the core features of the impulsive type, anger and affective instability characterize the aggressive type, abandonment avoidance and affective instability are the most prominent features of the dependent type, and identity disturbance and emptiness characterize the empty type.

While adolescent dimensions of borderline personality disorder were excluded from the present systematic review, a review of research in this area reveals a lack of agreement regarding the presence or number of borderline dimensions as seen in studies of adult BPD. Five studies [39–43] support a unidimensional model, while three studies suggest a two-dimensional model. Speranza, Pham-Scottez, Revah-Levy et al. [44] identify two dimensions based on internally or externally oriented traits, Haltigan & Vaillancourt [45] name an intra- and inter-personal factor and an impulsivity/reactivity factor, and Chabrol, Montovany, Callahan et al. [46] identify a painful feelings and an impulsivity factor. Three studies support a four-factor model [47–49]. Becker et al. ‘s [47] components are self-negation, irritability, poorly modulated relationships, and impulsivity. Leung & Leung[48] identify affect dysregulation, impulsivity, interpersonal disturbances, and self/cognitive dimensions. Finally, Bibi et al.’s [49] factor analysis resulted in the extraction of four factors, of which only two showed good values of internal consistency. Six dimensions were identified by Chabrol et al.[50].

Another subset of empirical research investigating the structure of BPD, not included in the present review, aimed to establish the factor structure of multiple personality disorders, in order to test the validity of the DSM clustering of personality disorders [51–57], resulting in conflicting findings. Some of the studies are in favor of the diagnostic construct of BPD, and its placement in Cluster B, but point to a small number of BPD criteria loading on another PD factor, or vice versa [51, 53, 54], while others [52, 55–57], propose an alternative grouping of PD criteria. It should be noted, that in Sharp et al.’s [55] study, the BPD criteria loaded so heavily on a general social and occupational dysfunction factor, that a specific BPD factor could not be extracted, confirming authors’ hypothesis that the BPD criteria represent a general dysfunction rather than a specific type of personality.

The present systematic review was conducted to explore the current research on the dimensions of BPD in adults, critically appraise the similarities and differences between the studies presented, and synthesize their conclusions in order to advance the understanding of BPD. The present systematic review examined the methods used to explore underlying BPD dimensions, the number of factors extracted by studies, the level of agreement accomplished in BPD dimension research, and the detection of common criteria clusters. To the best of our knowledge, this is the first systematic review following the PRISMA guidelines to summarize the research conducted in this area.

## Method

This systematic review conforms to the guidelines of the Preferred Reporting Items for Systematic Reviews and Meta-Analyses-PRISMA guidelines [58]. Ethical approval was not required.

## Inclusion and Exclusion Criteria

Eligible studies had to meet the following inclusion criteria: an original study investigating the dimensions of borderline personality disorder using item-level factor analysis of the DSM criteria, measured by a valid instrument adhering to the DSM criteria, published in a peer-reviewed journal. Only English language papers were considered.

Studies were excluded if they investigated borderline dimensions in adolescents or if they investigated the factor structure of multiple psychiatric diagnoses, multiple personality disorders, or multiple theoretical or clinical constructs. Cluster analytic studies exploring the heterogeneity of BPD from a person-centered approach were excluded. To ensure that the included studies measured the same diagnostic construct, studies using a screening measure that did not strictly follow the content and structure of the DSM criteria, were excluded. Given the comparative aim of this review, studies that used confirmatory factor analysis of only one model were also excluded, as it would not be possible to compare the fit of different models, although these studies are mentioned in the discourse section.

Reviews, case studies, opinion pieces, and qualitative studies were also excluded. There was no time limit set on searches in order to capture early data regarding BPD dimensions.

## Search Strategy

A total of 3072 articles were identified and stored in the reference management program Zotero, in the search conducted in June 2023 and concluded on the 15th of June, 2023. Three computerized databases were searched (Pubmed, Scopus, PsycNET). A further two articles were identified by searching for citations.

The search terms were: ("borderline personality disorder" OR "borderline personality disorder criteria" OR "borderline personality disorder symptoms") AND ("exploratory factor analysis" OR "confirmatory factor analysis" OR "principal component analysis" OR "factor analysis" OR dimension*).

Inclusion at the title/abstract stage was conservative (leaning towards inclusion) to ensure that all relevant articles were included.

## Results

### Study Selection

The database search identified a total of 3072 reports. After the removal of duplicates, the number was reduced to1764. Title screening led to the exclusion of 766 articles whose titles were clearly not relevant to the topic. In the next step, 998 studies were abstract-screened, of which 515 reports were considered to be clearly not relevant to the topic, and 195 were not quantitative research (reviews, opinion pieces, case, qualitative studies, etc.). 198 articles were sought for retrieval, of which 4 were not accessible, 7 were written in a language other than English, a further 7 were not published in a peer-reviewed journal, and one was not original quantitative research. The full text of 179 articles was assessed. Of these, 55 did not explore BPD heterogeneity using factor analysis, 14 were factor analytic studies in adolescents, 32 performed factor analysis of multiple psychiatric or psychological constructs, 27 used measures that did not adhere to the DSM criteria, 15 used cluster analytic methods to explore BPD heterogeneity using a person-centered approach, 8 performed confirmatory factor analysis testing only 1 model, and 2 were written in a language other than English.

A total of 6 studies were identified through citation searches and were fully assessed for eligibility. Of these, two were not factor analytic studies, two investigated the structure of multiple constructs, and one tested only one model.

**Fig. 1:**
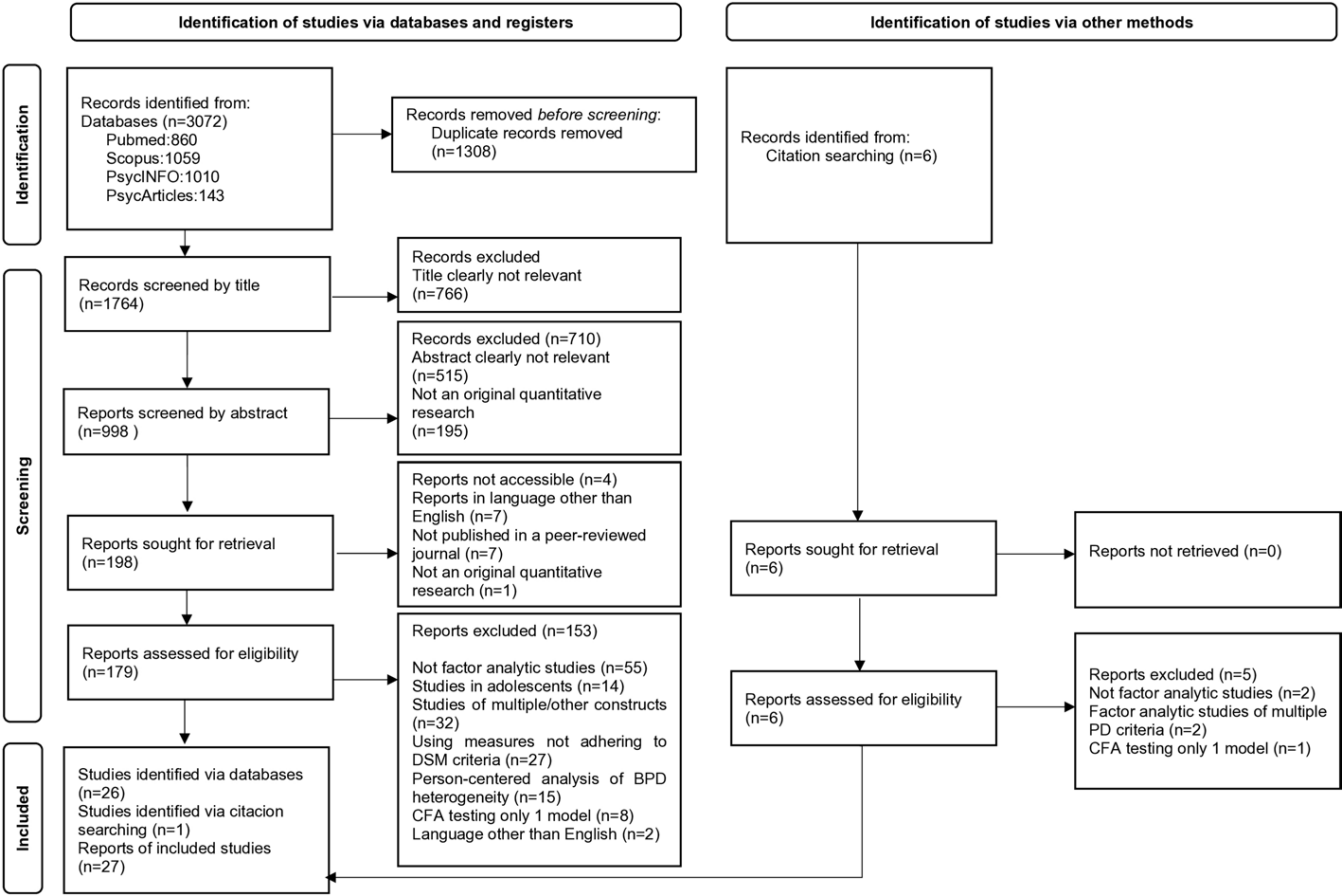
PRISMA diagram detailing the study selection process

A total of 27 studies of BPD dimensions identified by factor analysis were included in the present review [Fig. 1].

### Study Characteristics

The 27 studies included in the data extraction process, represent a total of 62,706 participants. Of these, 20,010 participated in a university group data depository [33] and 34,653 participated in the National Epidemiological Research NESARC [9]. 25 studies reported the gender of the participants, the majority of whom were women (mean = 63.62%, range = 19.4%−100%). 20 studies reported the mean age of participants, with a mean age of 29.71 years.

The majority of studies were conducted in the United States of America (*k* = 15), followed by Spain (*k* = 3). Research teams from Italy and the United Kingdom each conducted two studies, and from Norway, Canada, Australia, Singapore and Iran each conducted one study.

In terms of the sample used, 10 studies used a mixed clinical sample, 7 studies used a sample consisting only of people with BPD, 3 studies used a mixed community and clinical sample, and 7 studies used a community sample. Study characteristics and principal findings are depicted in Table 1.

**Table 1:** Study characteristics and principal findings

Author(s)/(Year)/ Country/ Title	Participants and setting	Method and data collection	Principal findings
Rosenberger & Miller (1989) USA [59] Comparing borderline definitions: DSM-III borderline and schizotypal personality disorders	106 undergraduates with psychopathological characteristics (56% female, 17% met BPD criteria)	Principal component analysis with Varimax rotation of DSM-III [60] criteria (SIDP)	2 factors accounting for 56% the of variance: 1. *Interpersonal disturbance* (38.9% of the var.) [factor loadings > .50: loneliness intolerance, identity disturbance, relationship instability] 2. *Instability* (16.7% of the variance) [factor loadings > .50: inappropriate anger, self-destruction, impulsivity, emotional liability]
Rusch, Guastello & Mason (1992) USA [30] Differentiating symptom clusters of borderline personality disorder	89 hospitalized patients with BPD (82% female, mean age = 27.8)	Principal axis factor analysis of DSM-III-R [61]criteria (clinical judgement)	3 factors with acceptable eigenvalue accounting for 48.3% of the variance: 1. *Self-Destructive unpredictability* (14.3% of the variance) [loadings > .50: affective instability, self-mutilating acts] 2. *Volatily factor* (17.2% of the variance) [loadings > .50: inappropriate anger, unstable interpersonal relationships, impulsivity] 3. *Identity disturbance* (16.9% of the variance) [identity disturbance] Abandonment fear loaded separately eig. < 1
Clarkin, Hull & Hurt (1993) USA [62] Factor structure of borderline personality disorder criteria	75 hospitalized females with a BPD diagnosis (mean age = 28)	Principal component with varimax rotation, using DSM-III-R criteria (SCID-II)	a. 3 factors accounting for 56% of the variance 1. *Identity problems/consequences in interpersonal relationships* (eig. = 2.09) [loadings > .50: emptiness, identity problems, fear of abandonment, unstable relationships] 2. *Emotional dimension* (eig. = 1.34) [factor loadings > .50: suicidal ideation, anger, emotional instability] 3. *Impulsivity* (eig. = 1.04) [impulsivity (0.90), negative correlation with emotional instability (−0.36)] b. 4 factors with eigenvalue > 1 accounting for 69% of the variance 1.*Fear of abandonment* (eig. = 2.09) [loadings > .50: fear of abandonment, emptiness, unstable relations] 2.*Labile Mood* (eig. = 1.35) [loadings > .50: labile mood, suicidality] 3.*Anger* (eig. = 1.04) 4. *Impulsivity* (eig. = 1.01)
Blais, Hilsenroth & Castlebury (1997) USA [63] Content validity of the DSM-IV borderline and narcissistic personality disorder criteria sets	91 people with a PD diagnosis (48% female, 21.9% with BPD, mean age = 28)	Principal component factor analysis with varimax rotation, of DSM-IV [64] criteria (clinical assessment)	3-factor solution accounting for 56% of the variance 1. *Interpersonal instability* (28% of variance, eig = 2.5) [loadings > .50: abandonment avoidance, unstable relationships, identity disturbance, emptiness] 2. *Affective and cognitive instability* (17% of variance, eig = 1.5) [ loadings > .50: inappropriate anger, stress related paranoia, affective instability] 3. Impulsivity related to affective and interpersonal instability (11% of variance, eig = 1.3) [loadings > .50: impulsivity, self-mutilating and suicidal behavior, unstable relationships, affective instability]
Fossati, Maffe, Bagnato et al. (1999) Italy [65] Latent structure analysis of DSM-IV borderline personality disorder criteria	564 inpatients and outpatients (57,6% female 17.7% with BPD, mean age = 29.9)	Weighted least squares confirmatory factor analysis of DSM-IV criteria (SCID-II) testing 1 to 4-factor models	One factor solution the best fitting model (*χ*^2^ = 18.89, df = 27, P = .874) with factor loadings ranging from .703 (suicidal behavior) to .903 (unstable relationships)
Sanislow, Grilo & McGlashan (2000), USA [66] Factor analysis of the DSM-III-R borderline personality disorder criteria in psychiatric inpatients	141 inpatients (47% female, 44% with BPD, mean age = 22.4)	Principal component factor analysis with Varimax rotation and principal axis factoring of DSM-III-R criteria (PDE)	3 factors accounting for 57.2% of the variance 1. *Disturbed relatedness*, (31.9% of the variance, eig = 2.55), [loadings > .50: unstable relationships, identity disturbance, emptiness] 2.*Behavioral dysregulation*, (13.5% of the variance, eig = 1.08), [loadings > .50: impulsivity, suicidal/self-mutilating behavior] 3. *Affective dysregulation* (11.8% of the variance, eig = 0.95), [loadings > .50: affective instability, inappropriate anger], abandonment avoidance (0.491)
Whenell, Ryman, Bonanno & Heather (2000) UK [67] Does the ICD 10 classification accurately describe subtypes of borderline personality disorder?	288 individuals with BPD referred to a PD treatment center (63.9% female)	Principal component factor analysis with Varimax rotation of DSM-III-R criteria (STCPD)	2 factor solution accounting for 39.8% of the variance: 1. *Calm-internalizing factor* (25.1% of the variance, eig = 2.0) [loadings > .50: fear of abandonment, impulsivity, suicide/self-harm] 2. *Mood externalizing factor* (16.4% of the variance, eig = 1.3) [loadings > .50: moodiness, inaproppriate anger] Unstable personal relationships and identity disturbance loaded on both factors, while emptiness loaded on a separate factor. Three factors with eigenvalue. > 1
Sanislow, Grilo, Morey et al. (2002) USA [68] Confirmatory factor analysis of DSM-IV criteria for borderline personality disorder: findings from the collaborative longitudinal personality disorders study	Mixed clinical sample of 668 participants (72.9% female, 35.9% with BPD, age range = 18–45)	Confirmatory factor analysis testing 1 and 3-factor (Sanislow et al., 2000) models, of DSM-IV criteria (DIPD-IV)	1- and 3-factor models both confirmed. Chi-squared test favored the 3-factor model. (χ^2^ = 8.1, df = 3, p < 0.05) 1. *Disturbed relatedness*, [loadings > .50: unstable relationships, identity disturbance, emptiness, stress-related paranoid ideation] 2. *Behavioral dysregulation* [loadings > .50impulsivity, suicidal or self-mutilating behavior] 3. *Affective dysregulation* [loadings > .50: affective instability, inappropriate anger, avoidance of abandonment] Factor correlations ranging between 0.94 and 0.99
Johansen, Karterud, Pedersen et al. (2004) Norway [14] An investigation of the prototype validity of the borderline DSM‐IV construct	930 patients of day treatment center (71.7% female, 27.1% with BPD, mean age = 34.6)	Confirmatory factor analysis testing 1-factor and 3-factor models (Sanislow et al., 2002) using DSM-IV criteria (SCID-II)	The 1-factor model was selected. The 3-factor model was also confirmed but not selected due to the high correlations between factors (0.94–1.00)
Benazzi (2006) Italy [69] Borderline personality–bipolar spectrum relationship	209 external patients with mood disorders	Principal component factor analysis with Varimax rotation, of DSM-IV criteria (SCID-II)	2 orthogonal factors accounting for 43.1% of the variance: 1.*Affective instability* (30.7% of the variance, eig = 2.7) [loadings > .50: unstable mood, unstable relationships, unstable self-image, emptiness, anger] 2. *Impulsivity* (12.4% of the variance, eig = 1.1) [loadings > .50: impulsivity, suicidal behavior, abandonment avoidance], paranoid ideation (0.42) No significant correlations between factors (*t* = 0.00, *p* = 1.000). The affective instability dimension was significantly correlated with bipolar disorder II
Clifton & Pilkonis (2007) [17] USA Evidence for a single latent class of Diagnostic and Statistical Manual of Mental Disorders borderline personality pathology	Mixed clinical and community sample of 411 people (64% female, 24.6% with BPD, mean age = 37.1)	Confirmatory factor analysis testing 1-factor model, 3-factor model (Clarkin et al., 1993), and 3-factor model (Sanislow et al., 2000;2002), using DSM-III-R criteria (clinical assessment)	1-factor model the best fit according to BIC. Factor loadings ranging from .44 to .74. All models were confirmed, high correlations between factors in the 3-factor models: 0.84–0.90 for Sanislow et. al. (2002) and 0.60–0.90 for Clarkin et al. (1993)
Feske, Kirisci, Tarter & Pilkonis (2007) USA [70] An application of item response theory to the DSM-III-R criteria for borderline personality disorder	353 mixed clinical sample (63.5% female, 77.1% psychiatric patiants, mean age = 36.9)	a. Exploratory factor analysis with varimax rotation of DSM-III-R or DSM-IV criteria (PDE, SIPD-IV, SCID-II) b. Confirmatory factor analysis testing 1-factor, 2-factor and 3-factor model (Sanislow et al., 2000) of DSM-III-R or DSM-IV criteria	a. 1 factor explaining 39.5% of the variance, (criteria loadings on the factor ranging from .53 to 0.78). Extraction of more components would result in components with eigenvalues < 1 b. Adequate fit for all three models. RMSEA only adequate fit the 3-factor model, AIC and BIC supported the 1-factor model. 1-factor model selected
Pérez, Barrachina, Soler et al. (2007) Spain [71] The clinical Clobal Impression Scale for borderline personality disorder patients (CGI-BPD): a scale sensible to detect changes	78 outpatients with BPD (85.9% female, mean age = 26.7)	Principal component analysis using DSM-IV-TR [72] criteria (CGI-BPD)	Two factors with eigenvalues > 1 explaining 67.4% of the variance 1. *Behavioral/interpersonal disorder*: [loadings > .50: Impulsivity, anger, suicide, paranoid ideation, unstable relationships] 2. *Problems of the self* [loadings > .50: identity, emptiness, abandonment, affect instability]
Taylor & Reeves (2007) USA [73] Structure of borderline personality disorder symptoms in a nonclinical sample	82 students with at least 1 BPD criterion present (63% female, 30.4% with BPD or sub-threshold BPD, mean age = 18.1)	Principal component analysis with Varimax rotation of DSM-IV criteria (SIDP-IV, SCID-II)	3 components solution accounting for 65.4% of the variance 1. *Self-other instability* (30.11% of the variance, eig = 2.71) [loadings > .50: abandonment avoidance, unstable relationships, identity disturbance, emptiness], suicidality/self-harm (.48) 2. *High affective instability* [.80] and low impulsivity [-.86] (18.14% of the var., eig = 1.63) 3. *Stress related paranoia* [.72] *and low impulsivity* [-.89] (17.18% of the variance, eig = 1.55) Negative correlations are explained in the context of sampling bias
Selby & Joiner, (2008) [74] USA Ethnic variations in the structure of borderline personality disorder symptomatology	Ethnically diverse sample of 1140 people in the community (45.2% female, age range = 18–23)	Principal component analysis with Varimax rotation of DSM-IV criteria (IPDE) on three groups: Caucasian (28% of the original sample), Hispanic (46%), African American (26%)	4-factor structure for all 3 ethnic groups accounting for approximately 70% of the variance 1. *Affective dysregulation* [anger, moodiness] 2. *Cognitive disturbance* [dissociation under stress, abandonment avoidance] 3. *Disturbed relatedness* [chaotic relationships] 4. *Behavioral dysregulation* [suicide and self-injury] The loading of the criteria of emptiness and impulsivity on a factor varied significantly between groups. Different primary factors across groups. Medium correlations between factors
Gardner & Qualter, (2009) UK [75] Reliability and validity of three screening measures of borderline personality disorder in a nonclinical population	Community sample of 523 individuals (77.7% female, mean age = 33.7)	a. Principal axis factor analysis using DSM-IV criteria (PDQ-4) b. Principal axis factor analysis using DSM-IV criteria (MSI-BPD)	a. PAF identified one factor with eigenvalue > 1 (3.58), confirmed by CFA b. PAF identified two factors with eigenvalue > 1 (4.54 and 1.003). CFA confirmed 1-factor model
Becker, Añez, Paris & Grilo, (2010) USA [76] Exploratory factor analysis of borderline personality disorder criteria in monolingual Hispanic outpatients with substance use disorders	130 monolingual Spanish-speaking individuals getting treatment for substance abuse (31% female, 30% with BPD, mean age = 37.4)	Principal axis factor analysis, using DSM-IV criteria (S-DIPD-IV)	1 factor accounting for 53% of the total variance with eigenvalue = 4.75. Factor loadings ranging from 0.53 (abandonment fears) to 0.77 (paranoia or dissociation)
Andión, Ferrer, Gancedo et al. (2011) Spain [77] Confirmatory factor analysis of borderline personality disorder symptoms based on two different interviews: the Structured Clinical Interview for DSM-IV Axis II Disorder and the Revised Diagnostic Interview for Borderlines	338 individuals (73.3% female, 65.1% with BPD, mean age = 27.2) referred to an outpatient BPD program	Confirmatory factor analysis testing 1-factor, 3-factor (Sanislow et al., 2002) and 5-factor model (Oldham, 2006), using DSM-IV criteria (SCID-II)	Sanislow’s (2002) 3-factor model was chosen by *χ*^2^ 1-factor model also confirmed. (*χ*^2^_diff_ = 16.09, *df* = 3, *p* < 0.05; *χ*^*2*^*:df* ratio = 1.54 vs. *χ*^*2*^*:df* ratio = 1.76; and BIC = 259.87 vs. BIC = 261.91) 1. *Disturbed relatedness*: [loadings > 0.50: paranoid ideation, emptiness, identity disturbance], unstable relationships (0.49) 2. *Behavioral dysregulation:* impulsivity (0.67), recurrent suicidal behavior (0.49) 3. *Affective dysregulation:* [loadings > .50: abandonment avoidance, affective instability], inappropriate anger (0.40) Correlations between factors ranging from 0.77–0.95
Chmielewski, Bagby, Quilty et al. (2011) Canada [78] A (re)-evaluation of the symptom structure of borderline personality disorder	373 students (19.4% female, mean age = 38.9) referred to a university psychiatric clinic	Confirmatory factor analysis testing 1 to 4-factor models of DSM-IV criteria (SCID-PQ)	Sanislow’s (2002) 3-factor model a better fit according to BIC and AIC, with medium correlations between factors (r = 0.59) Unidimensional model the worst fit
Eaton, Kkrueger, Keyes et al. (2011) USA [9] Borderline personality disorder co-morbidity: relationship to the internalizing–externalizing structure of common mental disorders	34,653 members of the community (48% female) participating in NESARC	Exploratory factor analysis of DSM-IV criteria (AUDADIS-IV)	The unidimensional model was favored by scree plot and fit indices, with factor loadings over .68. In a 2-factor solution, the factors were highly correlated (.83)
Calvo, Andión, Gancedo et al. (2012) Spain [79] Borderline Personality Disorder (BPD) diagnosis with the self-report Personality Diagnostic Questionnaire–4 + (PDQ-4 +): confirmation of the 3-factor structure	159 external patients of a psychiatric clinic diagnosed with BPD (76,7% female, mean age = 29.1)	Confirmatory factor analysis testing 1-factor and 3-factor (Sanislow et al., 2002) models, using DSM-IV criteria (PDQ-4)	Both models fitted the data. 3-factor model proved to be a better fit by *χ*^2^_diff_ (*χ*^2^_diff_ = 11.62; gl = 3; *p* = 0.01) Average to high between factors correlations (0.59 to 0.78)
Lewis, Caputi & Grenyer (2012) Australia [31] Borderline personality disorder subtypes: A factor analysis of the DSM‐IV criteria	95 diagnosed with BPD (86.3% female, mean age = 30)	Principal component analysis with oblique rotation, using DSM-IV criteria (SCID-II)	3-factor solution accounting for 57.8% of the variance 1. *Affect dysregulation* (21.4% of the variance) [loadings > .50: affective instability, inappropriate anger, impulsivity] 2. *Rejection sensitivity* (19.41% of the variance) [loadings > .50 suicidal behavior, abandonment avoidance, emptiness] 3. *Mentalization failure* (16.97% of the variance) [loadings > .50: stress-related paranoia, identity disturbance, negative] loading on unstable relationships (−0.69)
Hawkins, Furr, Arnold et al. (2014) USA [80] The structure of borderline personality disorder symptoms: a multi-method, multi-sample examination	281 individuals with BPD characteristics (30.6% with BPD, 68% women) in university psychiatric hospital	Principal axis factor analyses using DSM-IV criteria (SIDP-IV), (all participants, participants with BPD diagnosis and participants without)	1-factor structure supported in all-participants group. Lack of clear factorial structure for BPD group, results of non-BPD group partially support a one factor solution, though with 2 factors with eigenvalue > 1
Keng, Lee, Drabu et al. (2019) Singapore [81] Construct Validity of the McLean Screening Instrument for borderline personality disorder in two singaporean samples	Combined sample of 413. 124 inpatients and outpatients (female = 57.6, mean age 34.74, 36% BPD) and 289 undergraduates (female 72%, mean age = 19.89)	Confirmatory factor analysis testing 1-, 4- factor models (Leung et Leung, 2009), and modified 3-factor model using DSM-V [1] criteria (MSI-BPD)	*χ*^2^ showed better fit for the 3-factor model versus the 1-factor model for the combined sample (Δχ^2^ = 20.704, Δ*df* = 3, p = .0001). 4-factor model also a good fit 1. *Affect dysregulation* [loadings > .50: affect instability, anger dyscontrol] 2.*Self disturbance* [loadings > .50: dissociative symptoms, chronic emptiness, identity disturbance] 3.*Interpersonal and behavioral dysregulation* [loadings > .50: self-harm/suicide, impulsivity, paranoid ideation, unstable relationships, abandonment fears] Factor correlations ranging from .840 to .866 Results replicated for separate student and patient groups
Asl, Dabaghi & Taghva (2020) Iran [82] Screening borderline personality disorder: The psychometric properties of the Persian version of the McLean screening instrument for borderline personality disorder	254 soldiers (mean age = 25.71)	Confirmatory factor analysis testing 1 and 2-factor (Soler et al., 2016) [83] models using DSM-IV and DSM-5 criteria (MSI-BPD)	Both models fitted the data. Criteria loadings on 1 factor ranging from 0.54 (identity disturbance) to 0.83 (paranoid ideation) 2 factor model consisting of: 1.*Cognitive factor*: [loadings > .50: abandonment avoidance, emptiness], identity disturbance (0.49) 2.*Impulsivity factor*: [loadings > .50: unstable relationships, self-harm/suicide, impulsivity, affective instability, anger, paranoid ideation, dissociative symptoms] Factors correlation at 0.87
Johnson & Levy (2020) USA [33] Identifying unstable and empty phenotypes of borderline personality through factor mixture modeling in a large nonclinical sample	Sample of 20010 undergraduate students 63,86% female, mean age = 18.8)	a. Exploratory factor analysis using DSM criteria (MSI-BPD) b. Confirmatory factor analysis of 1 and 3-factors model (Johnson & Levy, 2020)	a. 3-factor solution accounting for 65.5% of the variance best fitted the data 1. *Affective/impulsive problems* [anger, affective instability, impulsivity, paranoia/dissociation] 2. *Emptiness/identity disturbance* 3. *Abandonment avoidance* Correlations between factors ranging from .65 to .81 b. Both 1 and 3-factors model fit the data. 3-factor model best fit (Δfit > .01)
Mneimme, Emery, Furr & Fleeson (2021) USA [84] Symptoms as rapidly fluctuating over time: Revealing the close psychological interconnections among borderline personality disorder symptoms via within-person structures	Mixed clinical and community sample of 252 individuals (29.3% with BPD, 67,5% female, mean age = 44)	Principal component analysis, using DSM-IV criteria, (SIDP-IV)	1-factor accounting for 66% of the variance, with factor loadings ranging from .55 to .75), confirmed by scree plot. Results replicated in subgroup of participants

Psychometric measures index: Alcohol Use Disorder and Associated Disabilities Interview Schedule—DSM-IV Version (AUDADIS-IV; Grant et al., 1995); Global Impression Scale for Borderline Personality Disorder (CGI-BPD; Perez et al., 2007), International Personality Disorder Examination (IPDE, Loranger et al., 1994); McLean Screening Instrument for BPD (MSI-BPD, Zanarini et al., 2003); Personality Disorder Examination (PDE, Loranger et al., 1987; Personality Disorder Questionnaire (PDQ, First, Spitzer, Gibbon, & Williams, 1997b); Screening Test for Comorbid Personality Disorder (STCPD; Dowson, 1992); Spanish-Language Version of the Diagnostic Interview for DSM-IV Personality Disorders (S-DIPD-IV; Grilo et al., 2003); Structured Clinical Interview for DSM-IV Axis II disorders (SCID-II: First, Spitzer, Gibbon, & Williams, 1997b); Structured Interview for the DSM-III Personality Disorders (SIDP; Pfohl, Stangl, & Zimmerman, 1983); Structured Interview DSM-IV Personality (SIDP-IV; Pfhol et al., 1997); The Diagnostic Interview for DSM-IV Personality Disorders (DIPD-IV). Belmont, MA: McLean Hospital)

The studies’ uality was assessed using the Appraisal Tool for Cross-Sectional Studies (AXIS [85]. According to AXIS criteria we examined whether the studies followed an appropriate study design to get meaningful results with low-bias. The overall quality did not vary significantly across studies, while the majority of studies were of moderate quality. One of the main quality issues was the use of convenience samples that did not represent the population, while some studies did not clearly define the population they investigated, mostly because they used mixed clinical and community samples. Regarding sample selection, all studies but one did not provide a justification for the sample’s size or follow a proper selection process where each member of the population has an equal chance of being included. The above, while resulting in reduced statistical power, reflects the challenges imposed by the research on clinical populations. Most studies did not declare competing interests, while seven studies did not address limitations. The majority of the studies met 10–15 AXIS criteria, while two studies met less than 10 AXIS criteria. Additional data regarding the studies’ appraisal are given in Online Resource 1.

### Methods Used by Studies Exploring Factor Structure

Factor analytic methods can be used to specify the dimensions of a given construct, in our case diagnostic, and therefore, most research on the dimensions underlying BPD is based on factor analysis. The two general classes of factor analysis are exploratory factor analysis and confirmatory factor analysis. In exploratory methods, there are no expectations about the number of factors, and even if there are, the analysis cannot be influenced by them, while the purpose of exploratory methods is to extract factors that can be interpreted. On the other hand, confirmatory factor analysis requires that the number of factors, latent variables, and factor correlations of the models tested are based on a preexisting model, as it tests the fit of previously proposed factor models [86].

18 of the studies included in this review used exploratory methods. Exploratory factor analysis (EFA) was used in 3 studies [9, 33, 70]. Principal component analysis, which is the most frequently used factor extraction method in EFA [86], was used by 11 studies [31, 59, 62, 63, 66, 67, 69, 71, 73, 74, 84], while principal axis factoring, another factor extraction method used in EFA, was used by 4 studies [30, 47, 75, 80]. Two studies [33, 70] used both exploratory and confirmatory analyses.

Confirmatory factor analysis (CFA) studies that test only one model were excluded from the present review, given the comparative aim of the review, and bearing in mind that, as mentioned by Thompson [86], one should not conclude that a model has been proven if one has not tested competing models, as a number of models could fit a given data set, especially if there are other theoretically or empirically derived models of the construct. This was particularly true for this review, as, out of the 11 studies that used CFA to test multiple models [14, 17, 33, 65, 68, 70, 77–79, 81, 82], 9 confirmed acceptable fit for more than one model [14, 17, 33, 68, 70, 77, 79, 81, 83].

The majority of studies supported multidimensional models. 13 studies using exploratory methods supported a multidimensional model [30, 31, 33, 54, 60, 62, 63, 67, 69, 71, 73, 74] while 5 studies supported the unidimensional model [70, 75, 76, 80, 84]. 5 studies using confirmatory factor analysis and testing more than one model supported a multidimensional model [68, 77–79, 81, 82], while 4 studies supported the unidimensional structure of BPD [14, 17, 65, 70]. One study [82], confirmed two models without selecting the best fit. The fit indices of the models tested by the studies using CFA are presented in Table 2. *According to these findings, exploratory methods, tended to extract more than one factor, and this inclination was consistent across different extraction methods used (PCA or PAF), while confirmatory analyses were divided regarding the support of unidimensional or multi-dimensional models*.

**Table 2:** Goodness of fit indices for tested models from studies using CFA

Fossati et al., 1999	*χ*^*2*^	*df*	*P*					
Unidimensional/ congenericity	18.89	27	.874					
Unidimensional/tau-equivalenc	80.30	35	< .001					
Unidimensional/parallelism	80.30	43	< .001					
Threedimensional (Clarkin et al., 1993)/ orthogonal factors	1858.31	24	< .001					
Three-dimensional (Hurt et al., 1990)/orthogonal factors	2889.5	25	< .001					
Four dimensional (Clarkin et al., 1993)/orthogonal factors	2713.85	24	< .001					
Sanislow et al., 2002	NFI	CFI	RMSEA					
1-factor model	0.947	0.960	0.066					
3-factor model (Sanislow et al., 2000)	0.951	0.963	0.067					
Johansen et al. (2004)	χ^2^	*df*	RMSEA	NFI	CFI	GFI	NNR	
1-factor model	78.434	27	0.0463	0.934	0.955	0.991	0.941	
3-factor model (Sanislow et al. 2000)	75.769	24	0.0492	0.936	0.955	0.992	0.933	
Clifton & Pilkonis, 2007	χ^2^	*df*	RMSEA	BIC	CFI			
1-factor /congeneric	68.8	20	0.077	165.1	0.934			
1-factor/ equivaleince	174.7	27	0.116	228.9	0.802			
1-factor/parallel	1347.7	35	0.302	1353.7	0			
3-factors (Clarkin et al., 1993)/ oblique	63.8	18	0.079	172.2	0.938			
3-factors (Clarkin et al., 1993)/ orthogonal	398.8	21	0.209	489.0	0.493			
3-factors (Sanislow et al., 2000;2002)/ oblique	59.0	17	0.078	173.3	0.944			
3-factors (Sanislow et al., 2000;2002)/ orthogonal	400.0	21	0.21	489.9	0.491			
Feske et al. (2007)	χ^2^	*df*	*p*	RMSEA	CFI	NFI	AIC	BIC
1-factor model	15.9	10	.10	.041	.99	.99	67.86	82.16
2-factor model (rationally divided)	14.9	9	.095	.043	.99	.99	68.86	83.71
3-factor model (Sanislow et al. 2000;2002)	13.1	7	.071	.050	.99	.99	71.05	87.00
Andión et al. (2011)	χ^2^	*df*	*p*	RMSEA	CFI	TLI	AIC	BIC
1-factor model	111.14	65	< 0.001	0.05	0.94	0.92	166.14	261.91
3-factor model (Sanislow et al., 2000, 2002)	95.05	62	< 0.05	0.04	0.96	0.95	153.05	259.88
Calvo et al. (2012)	χ^2^	*df*	RMSEA	TLI	CFI	AIC	GFI	
1-factor	53.14	27	0.078	0.813	0.860	89.137	0.939	
3-factor (Sanislow et al., 2000, 2002)	41.52	24	0.068	0.859	0.906	83.518	0.953	
Johnson & Levy (2019)	In(L)	*K*	CFI	AIC	BIC	BIC_adj_		
1-factor model	−65,298	18	1.00	130,632	130,76	130,72		
3-factor model (Johnson & Levy, 2019)	−64,605	33	.98	129,277	129,54	129,43		
Keng et al. (2019)	χ^2^	*df*	CFI	RMSEA	TLI			
1-factor	86.543	35	.981	.059	.976			
3-factor (modified Leung & Leung, 2019) model	53.760	32	.992	.040	.989			
4-factor model (Leung et Leung, 2019)	53.287	29	.991	.045	.986			
Asl et al. (2020)	χ^2^	*df*	RMSEA	NFI	GFI	TLI	RFI	AGFI
1-factor	106	35	0.08	0.88	0.92	0.89	0.85	0.88
2-factor (Soler et al., 2016)	97	34	0.08	0.89	0.93	0.90	0.85	0.88
Chmielewski et al. (2011)	In(L)	*K*	AIC	BIC				
Single factor	–2191.76	30	5223.527	5341.174				
2factor/ (Rosenberger et al., 1989)	–2572.33	31	5206.66	5328.229				
2factor/(Benazzi, 2006)	–2572.50	31	5207.007	5328.576				
3-factor/(Sanislow,2002)	–2552.22	33	5170.516	5299.928				
3-factor/(Clarkin et al., 1993)	–2570.62	33	5207.247	5336.659				
3-factor/(Taylor & Reeves, 2007)	–2571.50	33	5208.998	5338.41				
4-factor (Becker et al., 2006)	–2569.15	36	5210.294	5351.47				

*Fit indices: χ*^2^ minimum fit function chi-square *df* degrees of freedom, *RMSEA* root mean square error of approximation, *CFI* comparative fix index, *NFI* normed fit index, *AIC* Akaike information criteria, *TLI* Tucker-Lewis Index, *GFI* Goodness of fit intex, *In(L)* log-likelihood, *K *number of parameters, *NNFI* non-normed fit index, *GFI* Goodness of fit index, *NFI* Normed fit index, *NNFI* Non-NFI, *TLI* Tucker Lewis index, *RFI* Relative fit index, *AGFI* Adjusted goodness of fit index, *BIC* Bayesian information criterion

It should be noted that all 8 studies that used confirmatory factor analysis to test only one model, which were excluded from the present study, confirmed [18, 52, 87–90] or partially confirmed [91, 92] the one-dimensional model.

### Factor Structure in Multidimensional Models

#### Empirical Support and Factor Structure of Two-Dimensional Models

Five of the studies supported a two-dimensional model [59, 67, 69, 71, 83].With the exception of Asl et al. [82], the above-mentioned studies used exploratory methods. The first study on the dimensions of borderline personality disorder [59] identifies a disturbed relationships factor, including the criteria of emptiness and identity disturbance, and an impulsivity factor, including the criteria of self-mutilating behavior, anger, and affective instability. Whenell et al. [67] and Benazzi et al. [68] provide a similar structure, grouping fear of abandonment, impulsivity, and suicidal/self-mutilating behavior in one factor, and the criteria of affective instability and inappropriate anger in another factor, while Asl et al. [82] confirm a two-factor model [83], with more symptom clustering in the impulsivity factor. Similar to Asl et al.[82], Perez et al. ‘s [71] two-factor model identifies a *“behavioural/interpersonal disorder”* and a *“problems of the self”* factor.

The percentage of variance accounted for by a two-factor model was relatively low in studies using exploratory methods. Whenell et al. [67] factors account for 39.8% of the variance, Benazzi et al.’s [69] factors account for 43.1% of the variance, while Rosenberger & Miller’s [59] factors, account for 56% of the variance. In general, the two-dimensional models appear to support a structure formed around the poles of impulsivity and affective instability, consistent with the ICD-10’s division of emotionally unstable personality disorder into a borderline and an impulsive subtype, with two of the studies [67, 69] concluding that their results support the ICD-10’s EUD subtyping.

In terms of criterion groupings, it appears that identity disturbance and chronic feelings of emptiness are linked closely across studies, as core elements of what is described in the ICD-10 as the borderline subtype, and that another grouping is formed around marked impulsivity and suicidal/self-harming behaviors, while the other criteria do not consistently form groupings [Appendix 1].

#### Empirical Support and Factor Structure in 3-Dimensional Models

A total of 12 studies supports a three-dimensional model for the BPD construct [30, 31, 33, 62, 63, 66, 68, 73, 77–79, 81]. Seven of these studies used exploratory methods [31, 33, 62, 63, 66, 69, 73].The percentage of variance accounted for by a three-factor model in studies using exploratory methods ranges from 48.3% [30] to 65.5% [33]. The factors in the model of Sanislow et al. [66] account for 57.2% of the variance. Three-dimensional models were confirmed using CFA in five studies [68, 77–79, 81].

The most influential among the three-factor models is undoubtedly the model of Sanislow et al.[66, 68], which has been tested more than any other multidimensional model [14, 17, 70, 77–79, 93], and has been shown to be the best fit by three independent studies [77–79] and Sanislow et al.[68], while two other studies [14, 17] confirmed Sanislow’s 3-factor model, although the unidimensional model was a better fit. The stronger criticism of the model is that the correlations between the factors are high [14, 17, 80], suggesting a unidimensional structure of BPD. Nonetheless, the factor correlations of the model vary across studies, with Sanislow et al. [68] reporting high correlations ranging from 0.94 to 0.99, in line with Johansen et al. ‘s [14] 0.94-to 1.00 and Clifton & Pilkonis’s [17] 0.84–0.90. Andión et al. [77] found correlations between factors of from 0.77 to 0.95, while Calvo et al. [79] report lower correlations of 0.59–0.78, in line with Chmielewski et al., [78] who report insignificant correlations between factors (*r* = 0.59). Sanislow’s team [68] clarifies that the results of their study support the structure of BPD as a unidimensional construct consisting of three underlying dimensions (disturbed relatedness, behavioral dysregulation, affective dysregulation), and clarify that these dimensions do not necessarily imply the existence of subtypes, as the correlations between the factors are high.

With regard to the structure of the factors highlighted by the three-factor models, there is almost absolute agreement that the criteria of identity disturbance and chronic feelings of emptiness are found in the same dimension, suggesting a strong link between the two criteria, which are usually found as part of a disturbed relatedness dimension, in line with Sanislow et al.’s model [66, 68]. Some studies, also included stress-related paranoia/dissociation and abandonment avoidance in the above grouping, that might be described by Linehan’s [35]*“disorder of the self”* domain of borderline psychopathology. A close relationship is also observed, as was intuitively expected, between the inappropriate anger and affective instability criteria, which are usually grouped together under a dimension that could be called *“raging affect”*. The third pole appears to be a *“self-destructive”* pole, consisting of the suicidal/self-harm and impulsivity criteria, which are usually, but not always, grouped together. It should be noted that the fear of abandonment is not reliably associated with any of the other criterion groupings, which could at the same time mean that it is associated with all of the implied dimensions. In general, it can be observed that the grouping of the criteria follows the structure of Sanislow et al. [66, 68] while there is considerable agreement between the studies with regard to the factor structure. The loadings of the criteria on the three factor models, excluding the studies that confirm Sanislow et al. ‘s [66] findings, are presented in Appendix 2.

#### Empirical Support and Factor Structure of a Four-Dimensional Model

One study [74], provided support for a four-dimensional model of BPD, by examining the factor structure of the BPD criteria in an ethnically diverse sample. The factors were identified as affective dysregulation (anger, moodiness), cognitive disturbance (dissociation under stress, abandonment avoidance), disturbed relatedness (chaotic relationships), and behavioral dysregulation (suicide and self-injury). It is noted, that although all ethnic groups shared these dimensions, significant differences were observed in the factor loadings of chronic emptiness and inappropriate anger criteria on factors, a finding that is explained by cultural differences.

### Linking Dimensions to Subtypes

BPD heterogeneity has been explored through either variable-based or person-centered research, with no consensus on whether there is a clear link between dimensions and subtypes of the disorder. Researchers who support a unidimensional structure do not necessarily deny the possible existence of subtypes [17, 18, 65]. Fossati et al. [65] clarify that their research findings do not imply that BPD subjects could not be subtyped using variables external to the diagnostic criteria, in which case different therapeutic approaches might be beneficial to target different subtypes, sharing the point of view of Clifton & Pilkonis [17], who, having demonstrated the validity of BPD as a unidimensional construct, moved on to identify a single class of people with BPD, based on the fulfillment of the DSM criteria. However, these authors do not conclude that borderline subtypes do not exist, but that the search for such subtypes should move beyond the DSM criteria.

Later on, Hallquist & Pilkonis [18], while supporting the unidimensional structure through CFA, LCA,, and FacMM, substantiate their assumption of the existence of subtypes beyond the DSM criteria based on aggressive tendencies and anger expression, naming four subtypes of the disorder (angry/aggressive, angry/mistrustful, poor identity/low anger, prototypical). They note that the DSM criteria may identify the features of BPD that are shared between patients, but not those that differentiate among them.

On the other hand, researchers supporting multidimensional models, do not necessarily link the underlying dimensions to BPD subtypes. Despite confirming the most influential multidimensional structure, Sanislow et al. [68] note that this construct does not necessarily imply BPD subtypes, suggesting that the factors may reflect different aspects of the same disorder being present at different times. In contrast to Sanislow et al.[68], Calvo et al.[79], conclude that their findings which support Sanislow et al. ‘s structure of BPD, may imply the existence of disorder subtypes.

Two studies included in this review [67, 69], and one excluded study [93] conclude that their findings are consistent with ICD-10 ‘s [15] subtyping of the emotionally unstable disorder, based on their findings supporting a two-dimensional structure. In addition, Whenell et al. [67] identified four subtypes (calm-internalizing, mood-externalizing, combined, and undifferentiated), based on combinations of two factors (calm-internalizing and mood externalizing). The authors suggest adjusting the diagnostic system to include the four subtypes mentioned above.

Five of the studies examining the dimensions of the BPD criteria went on to differentiate subgroups of people diagnosed with BPD [30–33, 67], with their findings generally confirming that combinations of different dimensions correspond to specific subtypes. Rusch et al. [30] identify four subtypes (emotionally unstable, identity disturbed, emotionally disturbed and emotionally unstable, self-mutilating without emotional instability or identity disturbance), based on three dimensions (self-destructive unpredictability, volatility, identity disturbance), while Lewis, Caputi & Grenyer [31] identify three subgroups of individuals with BPD (emotionally dysregulated, rejection sensitive, experiencing mentalization failure), based on three corresponding dimensions.

The influential model of Sanislow et al. [66, 68] was studied regarding the existence of subtypes based on the three dimensions (disturbed relatedness, behavior dysregulation, and affective dysregulation) previously confirmed, in a sample of individuals referred to a specialized BPD program [32]. The study identified five classes of participants (a class of disturbed relatedness, a class of behavioral dysregulation, a class of affective dysregulation, a class in which all dimensions were present, and a class in which no dimensions were present). It should be noted that the largest class (40.8%) was that of participants meeting criteria for all three dimensions and that 36.2% of participants did not meet BPD criteria.

Johnson & Levy [33] studying a community sample with BPD elements, identified four classes (asymptomatic, affective/impulsive, empty/identity disturbed, “BPD” class), following their factor analytic findings of three BPD dimensions (affect, impulsivity, and paranoia/dissociation). Further analysis of their data using factor mixture modeling revealed a one-factor, three class (asymptomatic, empty, unstable) model, leading them to propose an “*unstable”* and “*empty*” phenotype of BPD.

## Discussion

The purpose of this review was to examine empirical studies and provide a synthesis of the research on the structure of the DSM diagnostic criteria for BPD. Research on the dimensions of BPD in adulthood has produced conflicting findings, although it appears that both the one-factor model and a three-factor model proposed by Sanislow et al. [66, 68] have gained significant empirical support. Inevitably, the question arises as to whether it is possible for the BPD construct to be both unidimensional and three-dimensional. Sanislow et al.[68], suggested to view one- and three-factor models as non-competing, arguing that BPD is a unified diagnosis based on three constituent components that can be used in order to clarify etiological pathways, better target problematic symptoms, and explain heterogeneity. Aiming to ensure comparability of the data, the present review did not include studies exploring the factor structure of the ICD-10’s [15] emotionally unstable disorder. While two-factor models generally support the ICD-10’s subtyping of EUD, further studies should explore similarities and discrepancies between the EUD and the BPD factor structure.

The clusters of criteria highlight the mixture of traits, symptoms, and symptomatic behaviors [5] that make up the diagnosis of BPD. From a trait perspective, the criteria of identity disturbance and chronic feelings of emptiness are almost inextricably linked, both in studies supporting a three-factor structure and in studies supporting a two-factor structure, while at the same time, three factor models link identity disturbance/emptiness to unstable relationships, and some two-factor models link identity disturbance/emptiness to affective instability. This strong association is consistent with Hurt et al. ‘s [34]*“identity cluster”*, formed from the criteria of emptiness, identity disturbance, and intolerance of being alone, and with Linehan’s [35]*“disorder of the self”* category composed of the same criteria. On the other hand, Gunderson’s [37] and Lieb et al.’s [36] suggestion to separate identity disturbance and chronic feelings of emptiness by placing emptiness in an “emotional dysregulation” domain, and identity disturbance in a “disturbed self” and “cognitive” domain, respectively, is not supported by empirical data. As the identity disturbance and chronic feelings of emptiness criteria are found to be the most consistently linked across studies, it is suggested that the understanding of this dimension should focus on the inability to form coherent descriptions of emotional and self-states rather than on interpersonal disturbance as suggested by Sanislow et al.[66, 68], and in this case, interpersonal disturbance could be seen as a direct consequence of this inability.

Another prominent grouping of criteria is that of behavioral symptoms, centered around impulsivity and self-harm/suicidal behavior in both three-factor and two-factor models. It should be noted that impulsivity and suicidal/self-harm behaviors are not consistently linked to any other criteria in three-dimensional models, whereas in two-dimensional models, impulsivity and suicidal/self-harm behavior are also linked to fear of abandonment. The above grouping is fully consistent with Hurt et al.’s [34] impulsive cluster, Linehan’s [35] behavioral dysregulation category, and Gunderson’s [37] and Lieb et al.’s [36] behavioral dyscontrol sector.

A grouping of affective symptoms is formed by inappropriate anger and affective instability, which are linked in the vast majority of three-factor and two-factor models, again consisted with Hurt et al.’s [34] affective cluster, Linehan’s [35] affective dysregulation category, and Gunderson’s [37] and Lieb et al.’s [36] emotional/affective dysregulation sector. This latter grouping also appears to partially support Akiskal’s [6] view of BPD as an affective disorder.

Regarding possible cultural differences in the structure of BPD, the majority of studies examining the factor structure of the disorder were conducted in the USA, while of the remaining studies, only two were conducted in non-western cultures [81, 82], with the Singaporean study [81] confirming a variation of the 3-factor model, and the Iranian study [82] supporting a two-factor structure. Only one study [74] examined the factor structure of BPD in an ethnically diverse sample, with the authors noting that while all groups shared the same dimensions, symptoms, possibly affected by cultural factors, loaded on different dimensions of the disorder. Given the different expression of symptoms across cultures, the results of this review regarding the factor structure of BPD could not be generalized for non-western cultures.

Differences are also observed depending on the sample use to determine the factor structure of BPD. It is observed, that all seven studies that used a sample of individuals meeting the BPD criteria [30, 31, 62, 63, 67, 71, 79] supported a multidimensional model, with the majority of them supporting a variation of the 3-factor structure. In contrast, studies using an exclusively community sample [9, 33, 73–75, 78, 82] tended to favor a one-factor structure to a greater degree than the studies using mixed or clinical samples, with three out of seven studies supporting the unidimensional model [9, 75, 82].

Regarding limitations of the study field of BPD dimensions and future directions of research, borderline dimensions and borderline subtypes are complimentary aspects of understanding borderline heterogeneity, and future research should further synthesize finding from both areas of research. Current research has linked borderline dimensions or combinations of dimensions to the existence of subtypes [30–33, 67], or provided support for ICD-10 [14] subtyping of emotionally unstable disorder [67, 69, 92]. Given the conflicting findings on the association between dimensions and subtypes, it seems that borderline heterogeneity can only be assessed by exploring BPD traits that go beyond the narrow boundaries of the DSM, focusing on the differences between individuals with the disorder, while exploring the association of BPD dimensions with BPD subtypes. From a treatment perspective, exploring BPD subtypes would likely help to individualize treatment for people with the disorder, while uncovering the underlying dimensions of the disorder would help to specifically target symptom clusters. The single-factor structure, having gained support from a number of studies [14, 17, 18, 52, 65, 70, 87–90], should also be examined in connection to specific BPD subtypes, while different treatment protocols could benefit those that conform to the unidimensional model. Another future direction of research could include the study of the stability of the criteria factor structure through time, as no studies with longitudinal data were detected in the present review, while it is possible that more externalizing traits, for example impulsivity, could remise through time, affecting the structure of BPD.

## Electronic supplementary material

Below is the link to the electronic supplementary material.

Supplementary file1 (PDF 187 KB)

## Appendix 1

Table 3.

**Table 3:** Criteria loading on two-dimensional models

Criteria	Abandonment avoidance	Unstable relationships	Identity disturbance	Impulsivity	Suicida l/ self-harm	Affective instability	Emptiness	Inappropriate anger	Paranoia/ dissociation
Study					Behavior				
Rosenberger & Miller, 1989		A	A	B	B	B	A	B	
Whenell et al., 2000	B			B	B	A		A	
Benazzi, 2006	B	A	A	B	B	A	A	A	B
Perez et al., 2007	A	B	A	B	B	A	A	B	B
Asl et al., 2010	A	B	A	B	B	B	A	B	B

Criteria loading on a common factor are depicted as sharing the same letter

## Appendix 2

Table 4.

**Table 4:** Criteria loading on 3-factor models of BPD

Criteria	Abandonment fear	Unstable relationships	Identity disturbance	Impulsivity	Suicidal/ self-harm	Affective instability	Emptiness	Inappropriate anger	Paranoia/ dissociation
Study					Behavior				
Rusch et al., 1992		Β	Α	Β	C	C		Β	
Clarkin, Hull & Hurt, 1993	Α	Α	Α	C	Β	Β	Α	Β	
Blais, Hilse nroth & Castlebury, 1997	Α	Α	Α	C	C	Β	Α	Β	Β
		C				C			
Sanislow, Grilo & McGlashan, 2000	Β	Α	Α	C	C	B	Α	Β	A
Taylor & Reeves, 2007	Α	Α	Α	-Β		Β	Α	-C	C
Lewis, Caputi & Grenyer, 2012	C	-Α	Α	Β	C	Β	C	Β	Α
Keng et al., 2019	C	C	A	C	C	B	A	B	A
									C
Johnson & Levy, 2020	C		Α	Β		Β	Α	Β	Β

Criteria loading on a common factor are depicted as sharing the same letter

## References

[1] American Psychiatric Association. Diagnostic and statistical manual of mental disorders (5th ed.). Washington, DC: Author. 2013. 10.1176/appi.books.9780890425596

[2] Stern Α. Psychoanalytic investigation of and therapy in the border line group of neuroses. Psychoanal Q. 1938;7(4):467–89. 10.1080/21674086.1938.11925367.

[3] Gunderson JG, Singer MT. Defining borderline patients: an overview. Am J Psychiatry. 1975. 10.1176/ajp.132.1.1. 802958 10.1176/ajp.132.1.1

[4] Spitzer RL, Endicott J, Gibbon M. Crossing the border into borderline personality and borderline schizophrenia: The development of criteria. Arch Gen Psyciatry. 1979;36(1):17–24. 10.1001/archpsyc.1979.01780010023001. 10.1001/archpsyc.1979.01780010023001 760694

[5] Sanislow CA, McGlashan TH. Treatment outcome of personality disorders. Can J Psychiatry. 1998;43(3):237–50. 10.1177/070674379804300302. 9561312 10.1177/070674379804300302

[6] Akiskal HS. Demystifying borderline personality: critique of the concept and unorthodox reflections on its natural kinship with the bipolar spectrum. Acta Psychiatr Scand. 2004;110(6):401–7. 10.1111/j.1600-0447.2004.00461.x. 15521823 10.1111/j.1600-0447.2004.00461.x

[7] Tyrer P. Primary article for discussion why borderline personality disorder is neither borderline nor a personality disorder. Personal Ment Health. 2009;3(2):86–95. 10.1002/pmh.78.

[8] Krueger RF. The structure of common mental disorders. Arch Gen Psyciatry. 1999;56(10):921–6. 10.1001/archpsyc.56.10.921. 10.1001/archpsyc.56.10.921 10530634

[9] Eaton NR, Krueger RF, Keyes KM, Skodol AE, Markon KE, Grant BF, Hasin DS. Borderline personality disorder co-morbidity: relationship to the internalizing–externalizing structure of common mental disorders. Psychol Med. 2011;41(5):1041–50. 10.1017/S0033291710001662. 20836905 10.1017/S0033291710001662 PMC3193799

[10] Markon KE. Modeling psychopathology structure: A symptom-level analysis of Axis I and II disorders. Psychol Med. 2010;40(2):273–88. 10.1017/S0033291709990183. 19515267 10.1017/S0033291709990183

[11] Kotov R, Ruggero CJ, Krueger RF, Watson D, Yuan Q, Zimmerman M. New dimensions in the quantitative classification of mental illness. Arch Gen Psyciatry. 2011;68(10):1003–11. 10.1001/archgenpsychiatry.2011.107. 10.1001/archgenpsychiatry.2011.107 21969458

[12] Bailey AJ, Finn PR. Borderline personality disorder symptom comorbidity within a high externalizing sample: relationship to the internalizing-externalizing dimensional structure of psychopathology. J Pers Disord. 2020;34(6):814. 10.1521/pedi_2019_33_415. 30730780 10.1521/pedi_2019_33_415 PMC7282285

[13] Skodol AE, Gunderson JG, Pfohl B, Widiger TA, Livesley WJ, Siever LJ. The borderline diagnosis I: psychopathology, comorbidity, and personaltity structure. Biol Psychiatry. 2002;51(12):936–50. 10.1016/S0006-3223(02)01324-0. 12062877 10.1016/s0006-3223(02)01324-0

[14] Johansen M, Karterud S, Pedersen G, Gude T, Falkum E. An investigation of the prototype validity of the borderline DSM-IV construct. Acta Psychiatr Scand. 2004;109(4):289–98. 10.1046/j.1600-0447.2003.00268.x. 15008803 10.1046/j.1600-0447.2003.00268.x

[15] World Health Organization. International statistical classification of diseases and related health problems. 10th ed. 2016. Retrieved June 10, 2023 from https://icd.who.int/browse10/2016/en.

[16] World Health Organization. International statistical clasiffication of diseases and related health problems. 11th ed. Retrieved June 10, 2023 from https://icd.who.int/browse/2025-01/mms/en#1128733473.

[17] Clifton A, Pilkonis PA. Evidence for a single latent class of Diagnostic and Statistical Manual of Mental Disorders borderline personality pathology. Compr Psychiatry. 2007;48(1):70–8. 10.1016/j.comppsych.2006.07.002. 17145285 10.1016/j.comppsych.2006.07.002

[18] Hallquist MN, Pilkonis PA. Refining the phenotype of borderline personality disorder: Diagnostic criteria and beyond. Pers Disord: Theory Res Treat. 2012;3(3):228. 10.1037/a0027953. 10.1037/a0027953 PMC3569527 22823231

[19] Hoermann S, Clarkin JF, Hull JW, Levy KN. The construct of effortful control: An approach to borderline personality disorder heterogeneity. Psychopathol. 2005;38(2):82–6. 10.1159/000084815. 10.1159/000084815 15802946

[20] Sleuwaegen E, Claes L, Luyckx K, Berens A, Vogels C, Sabbe B. Subtypes in borderline patients based on reactive and regulative temperament. Pers Individ Dif. 2017;108:14–9. 10.1016/j.paid.2016.11.065.

[21] Rufino KA, Ellis TE, Clapp J, Pearte C, Fowler JC. Variations of emotion dysregulation in borderline personality disorder: a latent profile analysis approach with adult psychiatric inpatients. Boderline Personal Disord Emot Dysregul. 2017;4(1):1–9. 10.1186/s40479-017-0068-2. 10.1186/s40479-017-0068-2 PMC5569486 28852520

[22] Newhill CE, Vaughn MG, DeLisi M. Psychopathy scores reveal heterogeneity among patients with borderline personality disorder. J Forens Psychiatry Psychol. 2010;21(2):202–20. 10.1080/14789940903281157.

[23] Morse JQ, Hill J, Pilkonis PA, Yaggi K, Broyden N, Stepp S, ... Feske U. Anger, preoccupied attachment, and domain disorganization in borderline personality disorder. J Pers Disord. 2009;23(3): 240. 10.1521/pedi.2009.23.3.240 10.1521/pedi.2009.23.3.240 PMC2821703 19538080

[24] Leihener F, Wagner A, Haaf B, Schmidt C, Lieb K, Stieglitz R, Bohus M. Subtype differentiation of patients with borderline personality disorder using a circumplex model of interpersonal behavior. J Nerv Ment Dis. 2003;191(4):248–54. 10.1097/01.NMD.0000061150.38924.2A. 12695736 10.1097/01.NMD.0000061150.38924.2A

[25] Ryan K, Shean G. Patterns of interpersonal behaviors and borderline personality characteristics. Pers Individ Dif. 2007;42(2):193–200. 10.1016/j.paid.2006.06.010.

[26] Salzer S, Streeck U, Jaeger U, Masuhr O, Warwas J, Leichsenring F, Leibing E. Patterns of interpersonal problems in borderline personality disorder. J Nerv Ment Dis. 2013;201(2):94–8. 10.1097/NMD.0b013e3182532b59. 23364116 10.1097/NMD.0b013e3182532b59

[27] Wright AG, Hallquist MN, Morse JQ, Scott LN, Stepp SD, Nolf KA, Pilkonis PA. Clarifying interpersonal heterogeneity in borderline personality disorder using latent mixture modeling. J Pers Disord. 2013;27(2):125. 10.1521/pedi.2013.27.2.125. 23514179 10.1521/pedi.2013.27.2.125 PMC3607958

[28] Critchfield KL, Clarkin JF, Levy KN, Kernberg OF. Organization of co-occurring Axis II features in borderline personality disorder. Br J Clin Psychol. 2008;47(2):185–200. 10.1348/014466507X240731. 17845740 10.1348/014466507X240731

[29] Smits ML, Feenstra DJ, Bales DL, de Vos J, Lucas Z, Verheul R, Luyten P. Subtypes of borderline personality disorder patients: a cluster-analytic approach. Boderline Personal Disord Emot Dysregul. 2017;4(1):1–15. 10.1186/s40479-017-0066-4. 10.1186/s40479-017-0066-4 PMC5494904 28680639

[30] Rusch KM, Guastello SJ, Mason PT. Differentiating symptom clusters of borderline personality disorder. J Clin Psychol. 1992;48(6):730–8. 10.1002/1097-4679(199211)48:6%3c730::AID-JCLP2270480606%3e3.0.CO;2-V. 1452761 10.1002/1097-4679(199211)48:6<730::aid-jclp2270480606>3.0.co;2-v

[31] Lewis K, Caputi P, Grenyer BF. Borderline personality disorder subtypes: A factor analysis of the DSM-IV criteria. Personal Ment Health. 2012;6(3):196–206. 10.1002/pmh.1183.

[32] Andión Ó, Ferrer M, Calvo N, Gancedo B, Barral C, Di Genova A, ... Casas M. Exploring the clinical validity of borderline personality disorder components. Compr Psychiatry. 2013;54(1): 34–40. 10.1016/j.comppsych.2012.06.004 10.1016/j.comppsych.2012.06.004 22794943

[33] Johnson BN, Levy KN. Identifying unstable and empty phenotypes of borderline personality through factor mixture modeling in a large nonclinical sample. Pers Disord: Theory Res Treat. 2020;11(2):141. 10.1037/per0000360. 10.1037/per0000360 31545635

[34] Hurt SW, Clarkin JF, Widiger TA, Fyer MR, Sullivan T, Stone MH, Frances A. Evaluation of DSM-III decision rules for case detection using joint conditional probability structures. J Pers Disord. 1990;4(2):121. 10.1521/pedi.1990.4.2.121.

[35] Linehan MM. Cognitive-behavioral treatment of borderline personality disorder. Guilford Press; 1993.

[36] Lieb K, Zanarini MC, Schmahl C, Linehan MM, Bohus M. Borderline personality disorder. The Lancet. 2004;364(9432):453–61. 10.1016/S0140-6736(04)16770-6. 10.1016/S0140-6736(04)16770-6 15288745

[37] Gunderson JG. Revising the borderline diagnosis for DSM-V: An alternative proposal. J Pers Disord. 2010;24(6):694. 10.1521/pedi.2010.24.6.694. 21158594 10.1521/pedi.2010.24.6.694 PMC3147142

[38] Oldham JM. Borderline personality disorder and suicidality. Am J Psychiatry. 2006;163(1):20–6. 10.1176/appi.ajp.163.1.20. 16390884 10.1176/appi.ajp.163.1.20

[39] Michonski JD, Sharp C, Steinberg L, Zanarini MC. An item response theory analysis of the DSM-IV borderline personality disorder criteria in a population-based sample of 11-to 12-year-old children. Pers Disord: Theory Res Treat. 2013;4(1):15. 10.1037/a0027948. 10.1037/a0027948 22642465

[40] Sharp C, Ha C, Michonski J, Venta A, Carbone C. Borderline personality disorder in adolescents: evidence in support of the Childhood Interview for DSM-IV Borderline Personality Disorder in a sample of adolescent inpatients. Compr Psychiatry. 2012;53(6):765–74. 10.1016/j.comppsych.2011.12.003. 22300904 10.1016/j.comppsych.2011.12.003

[41] Sharp C, Steinberg L, Temple J, Newlin E. An 11-item measure to assess borderline traits in adolescents: refinement of the BPFSC using IRT. Pers Disord: Theory ResTreat. 2014;5(1):70. 10.1037/per0000057. 10.1037/per0000057 PMC10754293 24588063

[42] Babinski DE, Castagna PJ, Waschbusch DA. Preliminary investigation of the psychometric properties of the parent version of the borderline personality features scale for children (BPFS-P). J Pers Assess. 2021;103(5):602–12. 10.1080/00223891.2020.1835934. 33124913 10.1080/00223891.2020.1835934

[43] Carreiras D, Loureiro M, Cunha M, Sharp C, Castilho P. Validation of the Borderline Personality Features Scale for Children (BPFS-C) and for Parents (BPFS-P) for the Portuguese population. J Child Fam Stud. 2020;29:3265–75. 10.1007/s10826-020-01800-7.

[44] Speranza M, Pham-Scottez A, Revah-Levy A, Barbe RP, Perez-Diaz F, Birmaher B, Corcos M. Factor structure of borderline personality disorder symptomatology in adolescents. Can J Psychiatry. 2012;57(4):230–7. 10.1177/070674371205700406. 22480588 10.1177/070674371205700406

[45] Haltigan JD, Vaillancourt T. The Borderline Personality Features Scale for Children (BPFS-C): Factor structure and measurement invariance across time and sex in a community-based sample. J Psychopathol Behav Assess. 2016;38:600–14. 10.1007/s10862-016-9550-1.

[46] Chabrol H, Montovany A, Callahan S, Chouicha K, Ducongé E. Factor analyses of the DIB-R in adolescents. J Pers Disord. 2002;16(4):374–84. 10.1521/pedi.16.4.374.24123. 12224129 10.1521/pedi.16.4.374.24123

[47] Becker DF, McGlashan TH, Grilo CM. Exploratory factor analysis of borderline personality disorder criteria in hospitalized adolescents. Compr Psychiatry. 2006;47(2):99–105. 10.1016/j.comppsych.2005.07.003. 16490567 10.1016/j.comppsych.2005.07.003

[48] Leung SW, Leung F. Construct validity and prevalence rate of borderline personality disorder among Chinese adolescents. J Pers Disord. 2009;23(5):494–513. 10.1521/pedi.2009.23.5.494. 19817630 10.1521/pedi.2009.23.5.494

[49] Bibi H, Kazmi SF. Urdu translation and validation of 11-item measure to assess borderline personality features in Pakistani adolescents. SAGE Open. 2021;11(1). 10.1177/2158244020986157

[50] Chabrol H, Montovany A, Ducongé E, Kallmeyer A, Mullet E, Leichsenring F. Factor structure of the borderline personality inventory in adolescents. Eur J Psychol Assess. 2004;20(1):59–65. 10.1027/1015-5759.20.1.59.

[51] Huprich SK, Schmitt TA, Richard D, Chelminski I, Zimmerman MA. Comparing factor analytic models of the DSM-IV personality disorders. Pers Disord: Theory Res Treat. 2010;1(1):22. 10.1037/a0018245. 10.1037/a0018245 22448603

[52] Nestadt G, Hsu FC, Samuels J, Bienvenu OJ, Reti I, Costa PT Jr, Eaton WW. Latent structure of the Diagnostic and Statistical Manual of Mental Disorders, personality disorder criteria. Compr Psychiatry. 2006;47(1):54–62. 10.1016/j.comppsych.2005.03.005. 16324903 10.1016/j.comppsych.2005.03.005

[53] Warren JI, South SC. A symptom level examination of the relationship between Cluster B personality disorders and patterns of criminality and violence in women. Int J Law Psychiatry. 2009;32(1):10–7. 10.1016/j.ijlp.2008.11.005. 19064289 10.1016/j.ijlp.2008.11.005

[54] Blais MA, Malone JC. Structure of the DSM-IV personality disorders as revealed in clinician ratings. Compr Psychiatry. 2013;54(4):326–33. 10.1016/j.comppsych.2012.10.014. 23219361 10.1016/j.comppsych.2012.10.014

[55] Sharp C, Wright AG, Fowler JC, Frueh BC, Allen JG, Oldham J, Clark LA. The structure of personality pathology: Both general (‘g’) and specific (‘s’) factors? J Abnorm Psychol. 2015;124(2):387. 10.1037/abn0000033. 25730515 10.1037/abn0000033

[56] Trull TJ, Vergés A, Wood PK, Jahng S, Sher KJ. The structure of Diagnostic and Statistical Manual of Mental Disorders (text revision) personality disorder symptoms in a large national sample. Pers Disord: Theory Res Treat. 2012;3(4):355. 10.1037/a0027766. 10.1037/a0027766 PMC3779622 22506626

[57] Westen D, Shedler J. Revising and assessing Axis II, Part II: Toward an empirically based and clinically useful classification of personality disorders. Am J Psychiatry. 1999;156(2):273–85. 10.1176/ajp.156.2.273. 9989564 10.1176/ajp.156.2.273

[58] Page MJ, McKenzie JE, Bossuyt PM, Boutron I, Hoffmann TC, Mulrow CD, ... Moher D. The PRISMA 2020 statement: an updated guideline for reporting systematic reviews. Syst Rev, 2021;10(1):1–11. 10.1186/s13643-021-01626-4 10.1186/s13643-021-01626-4 PMC8008539 33781348

[59] Rosenberger PH, Miller GA. Comparing borderline definitions: DSM-III borderline and schizotypal personality disorders. J Abnorm Psychol. 1989;98(2):161. 10.1037/0021-843X.98.2.161. 2708659 10.1037//0021-843x.98.2.161

[60] American Psychiatric Association. Diagnostic and statistical manual of mental disorders (3rd ed.). Washington, DC: Author. 1980. 10.1176/appi.books.9780890420188.dsm-iii

[61] American Psychiatric Association. Diagnostic and statistical manual of mental disorders (3rd ed., text rev.). Washington, DC: Author. 1987. 10.1176/appi.books.9780890420188.dsm-iii-r

[62] Clarkin JF, Hull JW, Hurt SW. Factor structure of borderline personality disorder criteria. J Pers Disord. 1993;7(2):137–43. 10.1521/pedi.1993.7.2.137.

[63] Blais MA, Hilsenroth MJ, Castlebury FD. Content validity of the DSM-IV borderline and narcissistic personality disorder criteria sets. Compr Psychiatry. 1997;38(1):31–7. 10.1016/S0010-440X(97)90050-X. 8980869 10.1016/s0010-440x(97)90050-x

[64] American Psychiatric Association. Diagnostic and statistical manual of mental disorders (4th ed.). Washington, DC: Author. 1994. 10.1176/appi.books.9780890420614.dsm-iv

[65] Fossati A, Maffei C, Bagnato M, Donati D, Namia C, Novella L. Latent structure analysis of DSM-IV borderline personality disorder criteria. Compr Psychiatry. 1999;40(1):72–9. 10.1016/S0010-440X(99)90080-9. 9924881 10.1016/s0010-440x(99)90080-9

[66] Sanislow CA, Grilo CM, McGlashan TH. Factor analysis of the DSM-III-R borderline personality disorder criteria in psychiatric inpatients. Am J Psychiatry. 2000;157(10):1629–33. 10.1176/appi.ajp.157.10.1629. 11007717 10.1176/appi.ajp.157.10.1629

[67] Whewell P, Ryman A, Bonanno D, Heather N. Does the ICD 10 classification accurately describe subtypes of borderline personality disorder? Br J Med Psychol. 2000;73(4):483–94. 10.1348/000711200160679. 11140789 10.1348/000711200160679

[68] Sanislow CA, Grilo CM, Morey LC, Bender DS, Skodol AE, Gunderson JG, ... McGlashan TH. Confirmatory factor analysis of DSM-IV criteria for borderline personality disorder: findings from the collaborative longitudinal personality disorders study. Am J Psychiatry, 2002;159(2):284–290. 10.1176/appi.ajp.159.2.284 10.1176/appi.ajp.159.2.284 11823272

[69] Benazzi F. Borderline personality–bipolar spectrum relationship. Prog Neuropsychopharmacol Biol Psychiatry. 2006;30(1):68–74. 10.1016/j.pnpbp.2005.06.010. 16019119 10.1016/j.pnpbp.2005.06.010

[70] Feske U, Kirisci L, Tarter RE, Pilkonis PA. An application of item response theory to the DSM-III-R criteria for borderline personality disorder. J Pers Disord. 2007;21(4):418–33. 10.1521/pedi.2007.21.4.418. 17685837 10.1521/pedi.2007.21.4.418

[71] Pérez V, Barrachina J, Soler J, Pascual JC, Campins M, Puigdemont D, Alvarez E. The clinical global impression scale for borderline personality disorder patients (CGI-BPD): a scale sensible to detect changes. Actas Esp Psiquiatr. 2007;35(4):229. 17592784

[72] American Psychiatric Association. Diagnostic and statistical.manual of mental disorders (4th ed., text rev.). Washington, DC: Author. 2000. 10.1176/appi.books.9780890420249.dsm-iv-tr

[73] Taylor J, Reeves M. Structure of borderline personality disorder symptoms in a nonclinical sample. J Clin Psychol. 2007;63(9):805–16. 10.1002/jclp.20398. 17674400 10.1002/jclp.20398

[74] Selby EA, Joiner TE Jr. Ethnic variations in the structure of borderline personality disorder symptomatology. J Psychiatr Res. 2008;43(2):115–23. 10.1016/j.jpsychires.2008.03.005. 18433775 10.1016/j.jpsychires.2008.03.005

[75] Gardner K, Qualter P. Reliability and validity of three screening measures of borderline personality disorder in a nonclinical population. Pers Individ Dif. 2009;46(5–6):636–41. 10.1016/j.paid.2009.01.005.

[76] Becker DF, Añez LM, Paris M, Grilo CM. Exploratory factor analysis of borderline personality disorder criteria in monolingual Hispanic outpatients with substance use disorders. Psychiatry Res. 2010;178(2):305–8. 10.1016/j.psychres.2009.03.016. 20472296 10.1016/j.psychres.2009.03.016 PMC2902552

[77] Andión Ó, Ferrer M, Gancedo B, Calvo N, Barral C, Torrubia R, Casas M. Confirmatory factor analysis of borderline personality disorder symptoms based on two different interviews: the Structured Clinical Interview for DSM-IV Axis II Disorder and the Revised Diagnostic Interview for Borderlines. Psychiatry Res. 2011;190(2–3):304–8. 10.1016/j.psychres.2011.05.014. 21640387 10.1016/j.psychres.2011.05.014

[78] Chmielewski M, Bagby RM, Quilty LC, Paxton R, Ng SAM. A (re)-evaluation of the symptom structure of borderline personality disorder. Can J Psychiatry. 2011;56(9):530–9. 10.1177/070674371105600904. 21959028 10.1177/070674371105600904

[79] Calvo N, Andión Ó, Gancedo B, Ferrer M, Barral C, Di Genova A, ... Casas M. Borderline personality disorder (BPD) diagnosis with the self-report personality diagnostic questionnaire–4+(PDQ-4+): confirmation of the 3-factor structure. Actas Esp Psiquiatr, 2012:40(2):57–62. 22508070

[80] Hawkins AA, Furr RM, Arnold EM, Law MK, Mneimne M, Fleeson W. The structure of borderline personality disorder symptoms: a multi-method, multi-sample examination. Pers Disord: Theory Res Treat. 2014;5(4):380. 10.1037/per0000086. 10.1037/per0000086 PMC4197810 25314228

[81] Keng SL, Lee Y, Drabu S, Hong RY, Chee CY, Ho CS, Ho RC. Construct validity of the mclean screening instrument for borderline personality disorder in two singaporean samples. J Pers Disord. 2019;33(4):450–69. 10.1521/pedi_2018_32_352. 29949444 10.1521/pedi_2018_32_352

[82] Asl EM, Dabaghi P, Taghva A. Screening borderline personality disorder: The psychometric properties of the Persian version of the McLean screening instrument for borderline personality disorder. J Res Med Sci, 2020;25. 10.4103/jrms.JRMS_949_19 10.4103/jrms.JRMS_949_19 PMC7698379 33273942

[83] Soler J, Domínguez-Clavé E, García-Rizo C, Vega D, Elices M, Martín-Blanco A, Feliu-Soler A, Carmona C, Pascual JC. Validation of the Spanish version of the Mclean screening instrument for borderline personality disorder. Validación de la versión Española del McLean screening instrument for borderline personality disorder. Rev Psiquiatr Salud Ment. 2016;9(4):195–202. 10.1016/j.rpsm.2016.03.002. 27067102 10.1016/j.rpsm.2016.03.002

[84] Mneimne M, Emery L, Furr RM, Fleeson W. Symptoms as rapidly fluctuating over time: Revealing the close psychological interconnections among borderline personality disorder symptoms via within-person structures. J Abnorm Psychol. 2021;130(3):260. 10.1037/abn0000656. 33539116 10.1037/abn0000656 PMC8274974

[85] Downes MJ, Brennan ML, Williams HC, Dean RS. Development of a critical appraisal tool to assess the quality of cross-sectional studies (AXIS). BMJ Open. 2016;6(12):e011458. 10.1136/bmjopen-2016-011458. 27932337 10.1136/bmjopen-2016-011458 PMC5168618

[86] Thompson B. Exploratory and confirmatory factor analysis: Understanding concepts and applications. Washington, DC, 2004;10694(000). 10.1037/10694-000

[87] Huprich SK, Paggeot AV, Samuel DB. Comparing the personality disorder interview for DSM–IV (PDI–IV) and SCID–II borderline personality disorder scales: An item–response theory analysis. J Pers Assess. 2015;97(1):13–21. 10.1080/00223891.2014.946606. 25203418 10.1080/00223891.2014.946606

[88] Cooper LD, Balsis S, Zimmerman M. Challenges associated with a polythetic diagnostic system: criteria combinations in the personality disorders. J Abnorm Psychol. 2010;119(4):886. 10.1037/a0021078. 20919789 10.1037/a0021078

[89] Munawar K, Aqeel M, Rehna T, Shuja KH, Bakrin FS, Choudhry FR. Validity and reliability of the urdu version of the Mclean screening instrument for borderline personality disorder. Front Psychol, 2021;3487. 10.3389/fpsyg.2021.533526 10.3389/fpsyg.2021.533526 PMC8417833 34489768

[90] Conway C, Hammen C, Brennan PA. A comparison of latent class, latent trait, and factor mixture models of DSM-IV borderline personality disorder criteria in a community setting: Implications for DSM-5. J Pers Disord. 2012;26(5):793. 10.1521/pedi.2012.26.5.793. 23013346 10.1521/pedi.2012.26.5.793 PMC3460547

[91] Huczewska I, Didyk P, Rogoza R. From categorical diagnosis to dimensional assessment of borderline personality. Curr Issues Personal Psychol. 2019;7(4):355–60. 10.5114/cipp.2019.89674.

[92] Le Corff Y, Martin-Storey A, Touchette L, Lapalme M, Forget K. Validation of a French translation of the McLean screening instrument for borderline personality disorder, invariance across genders, and association with depression, trauma symptoms, and substance use among university students. J Pers Disord. 2021;35(4):605–17. 10.1521/pedi_2020_34_494. 33779280 10.1521/pedi_2020_34_494

[93] Lai CM, Leung F, You J, Cheung F. Are DSM-IV-TR borderline personality disorder, ICD-10 emotionally unstable personality disorder, and CCMD-III impulsive personality disorder analogous diagnostic categories across psychiatric nomenclatures? J Pers Disord. 2012;26(4):551. 10.1521/pedi.2012.26.4.551. 22867506 10.1521/pedi.2012.26.4.551

