# Supplementary material for: Underlying Dimensions of Borderline Personality Disorder: A Systematic Review of Factor Analytic Studies
Source: Psychiatr Q. 2025 Apr 5;96(4):751–85. doi: 10.1007/s11126-025-10141-x (PMC12647336; doi:10.1007/s11126-025-10141-x)
Supplement: Supplementary file 1 — Supplementary file1 (PDF 187 KB) [file 11126_2025_10141_MOESM1_ESM.pdf]

Online resource1. Quality assessment of the studies included in the systematic review based on the AXIS tool

ITEM	Rosenberger, 1989	Rusch, 1992	Clarkin, 1993	Blais, 1997	Fossati, 1999	Sanislow,2000	Whenell, 2000	Sanislow,2002	Johansen, 2004	Benazzi, 2006	Clifton, 2007	Feske, 2007	Pérez, 2007	Taylor, 2007	Selby, 2008	Gardner, 2009	Becker, 2010	Andión, 2011	Chmielewski, 2011	Eaton, 2011	Calvo, 2012	Lewis, 2012	Hawkins, 2014	Keng, 2019	Asl, 2020	Johnson, 2020	Mneimme, 2021
**Clearly stated objectives**	N	Y	Y	Y	Y	Y	Y	Y	Y	N	Y	Y	Y	Y	Y	Y	Y	Y	Y	Y	Y	Y	Y	Y	Y	Y	Y
**Appropriate study design**	Y	Y	Y	Y	Y	Y	Y	Y	Y	Y	Y	Y	Y	Y	Y	Y	Y	Y	Y	Y	Y	Y	Y	Y	Y	Y	Y
**Sample size justification**	N	N	N	N	N	N	N	N	N	N	N	N	N	N	N	N	N	N	N	Y	N	N	N	N	N	N	N
**Population clearly defined**	N	Y	Y	Y	Y	Y	Y	Y	Y	Y	N	Y	Y	Y	Y	Y	Y	Y	Y	Y	Y	Y	Y	N	Y	Y	N
**Representantive sample**	N	N	N	N	N	N	N	N	N	N	N	N	N	N	N	N	N	N	N	Y	N	N	N	N	N	N	N
**Proper selection process**	N	N	N	N	N	N	N	N	N	N	N	N	N	N	N	N	N	N	N	Y	N	N	N	N	N	N	N
**Address non-responders**	N	N	N	N	N	N	Y	N	N	N	N	N	N	N	N	N	N	N	N	N	N	N	N	N	N	N	N
**Appropriate measures**	Y	Y	Y	Y	Y	Y	Y	Y	Y	Y	Y	Y	Y	Y	Y	Y	Y	Y	Y	Y	Y	Y	Y	Y	Y	Y	Y
**Reliable measures**	Y	Y	Y	Y	Y	Y	Y	Y	Y	Y	Y	Y	Y	Y	Y	Y	Y	Y	Y	Y	Y	Y	Y	Y	Y	Y	Y
**Precision estimates**	Y	Y	Y	Y	Y	Y	Y	Y	Y	Y	Y	Y	Y	Y	Y	Y	Y	Y	Y	Y	Y	Y	N	Y	Y	Y	Y
**Sufficient methods description**	N	Y	Y	Y	Y	Y	Y	Y	Y	Y	Y	Y	Y	Y	Y	D	Y	Y	Y	N	Y	Y	Y	Y	Y	Y	Y
**Data adequately described**	Y	Y	Y	Y	Y	Y	N	Y	Y	N	Y	Y	Y	Y	N	Y	Y	Y	Y	N	Y	Y	N	Y	Y	Y	Y

**Non-responders information**	N	N	N	N	N	N	N	N	N	N	N	N	N	N	N	N	N	N	N	N	N	N	N	N	N	N	N
**Results internally consistent**	Y	Y	Y	Y	Y	Y	Y	Y	Y	Y	Y	Y	Y	Y	Y	D	Y	Y	Y	D	Y	Y	D	Y	Y	Y	Y
**Comprehensive description of results**	Y	Y	Y	Y	Y	Y	Y	Y	Y	Y	Y	Y	Y	Y	Y	Y	Y	Y	Y	N	Y	Y	Y	Y	Y	Y	Y
**Results justify conclusions**	Y	Y	Y	Y	Y	Y	Y	Y	Y	Y	Y	Y	Y	Y	Y	Y	Y	Y	Y	Y	Y	Y	N	Y	Y	Y	Y
**Limitations**	N	Y	Y	Y	Y	Y	N	Y	Y	Y	N	N	N	Y	Y	Y	Y	N	Y	Y	N	Y	Y	Y	Y	Y	Y
**Conflict of interest**	N	N	N	N	N	Y	N	Y	N	N	N	N	N	N	N	N	Y	Y	Y	Y	Y	N	N	N	Y	N	Y
**Ethics approval**	D	Y	Y	N	Y	Y	N	Y	N	N	Y	Y	N	Y	N	N	Y	Y	Y	Y	Y	Y	N	N	Y	Y	N
